# Post-Stroke Recrudescence: A Case Report and Literature Review

**DOI:** 10.7759/cureus.43461

**Published:** 2023-08-14

**Authors:** Manogna Sagiraju, Raghav Prasad, Milenko Lazarevic

**Affiliations:** 1 Internal Medicine, Kempegowda Institute of Medical Sciences, Bangalore, IND; 2 Neurology, Kempegowda Institute of Medical Sciences, Bangalore, IND; 3 Internal Medicine, Swedish Covenant Hospital, Chicago, USA

**Keywords:** post-stroke recrudescence, altered drug response, focal stroke deficits, stroke work-up, immune activation, stroke management, acute stroke mimic, transient neurological attack

## Abstract

Post-stroke recrudescence (PSR) is a clinical entity characterized by the acute transient recurrence of previously recovered focal stroke deficits. Various names have been used to describe PSR, which further complicates its diagnosis. Increased awareness of this condition is crucial for preventing inappropriate management and unnecessary testing. Mechanisms proposed for PSR include altered drug response in diseased brain areas and immune activation due to a compromised blood-brain barrier (BBB). Patients with PSR have a distinct vascular risk profile and fewer cardiovascular complications than those with transient ischemic attacks (TIAs). Accurate differentiation of PSR from other conditions that mimic stroke is essential for its appropriate management. Misdiagnosis may lead to unnecessary procedures and prolonged hospitalization. This article presents the case of a 56-year-old female with multiple episodes of PSR that were initially misdiagnosed in the emergency department. The patient had a history of hypertension and ischemic stroke, and her episodes of PSR were often triggered by elevated blood pressure. Future studies should focus on developing validated prediction scores to guide recurrent stroke workup. Enhancing awareness and understanding of PSR can optimize resource allocation and improve patient outcomes.

## Introduction

Post-stroke recrudescence (PSR) is an under-recognized clinical entity defined as an acute transient recurrence of previous but recovered focal stroke deficits. It is characterized by the following criteria: transient worsening of residual post-stroke focal neurologic deficits or transient recurrence of previous stroke-related focal neurologic deficits, identifiable stressors, chronic stroke on brain imaging, absence of acute lesions on diffusion-weighted imaging (DWI), and an unlikely alternative diagnosis [1].

There are several names given to the same clinical entity of PSR, such as recrudescence of old stroke deficits (ROSD) [2], differential awakening [3], re-emergence [4], anamnestic recall [5], exacerbation of focal neurological deficits [6], locus minoris resistentiae [7], and “metabolic insult causing reexpression of old stroke” [8], which adds to further confusion in terms of diagnosing the condition in clinical practice. Here, we chose PSR because it is concise and clear.

PSR is a relatively common phenomenon, affecting one in 10 patients with transient neurological attacks. In one study, although patients with PSR and transient ischemic attack (TIA) had similar baseline vascular risk factors, patients with PSR had significantly fewer cardiovascular complications during the 90-day follow-up than those with TIA [2]. Therefore, it is essential to differentiate it from mimics, such as acute stroke, TIA, migraine, and seizure, as these conditions have different management protocols.

The patient in the present case had several episodes of PSR, which were diagnosed differently each time in the emergency department. She also appeared to have elevated blood pressure as a trigger for episodes of PSR. Increased awareness of this condition might have prevented inappropriate diagnosis and testing of this patient.

## Case presentation

A 56-year-old Caucasian female with a past medical history of Addison’s disease, stroke, migraine, and hypertension presented with right-sided numbness and weakness.

Her last known well was at 09:00 on that day when she developed numbness in her right lower extremity, which progressed to her right upper extremity and the right side of the face. This was followed by weakness in the same region and slurring of the speech. With these symptoms, she presented to the emergency department at 15:30 am, which made her ineligible for thrombolytic therapy. The patient denied any loss of consciousness, drowsiness, confusion, headache, vision disturbances, vomiting, or fever.

The patient had a documented history of Addison's disease for which she was receiving hydrocortisone. She also had a history of two TIAs in 2016 and an ischemic stroke of the left middle cerebral artery (L MCA) in May 2017, which presented with right-sided weakness and paresthesia.

Since the stroke, she has reported multiple episodes that presented with symptoms similar to those of her prior stroke. The symptoms were equal or inferior in severity as compared to the initial stroke. During these episodes, the patient presented with elevated blood pressure, and following its reduction, her symptoms spontaneously resolved within a couple of hours. These episodes occurred on the following dates: August 15, 2017, May 30, 2018, August 31, 2021, July 26, 2022, and November 11, 2022. The patient's hypertension was treated with enalapril. 

Upon arrival at the emergency department, the patient's blood pressure was significantly elevated at 198/110 mmHg and her pulse rate was 110 bpm. The patient had a National Institutes of Health Stroke Scale (NIHSS) of 8. A neurological examination revealed a slight slurring of speech, with the rest of her higher mental functions being normal. Cranial nerve examination revealed facial asymmetry, reduced prominence of the right nasolabial fold, and weakness in the right trapezius and sternocleidomastoid muscles. Motor examination demonstrated reduced power on the right side, with the right upper extremity graded at 3/5 and the right lower extremity graded at 2/5, compared to a normal 5/5 strength on the left side. Reflexes were 2+ in all extremities, and Babinski and Hoffmann's signs were absent. Sensory examination revealed decreased sensation to light touch and vibration on the right side of the body, and hemineglect was absent. The patient exhibited a normal finger-nose-finger test and heel-shin test on the left side, whereas weakness precluded performing these tests on the right side. Right-sided pronator drift was observed. The results of the remaining systemic examinations were unremarkable.

**Figure 1:**
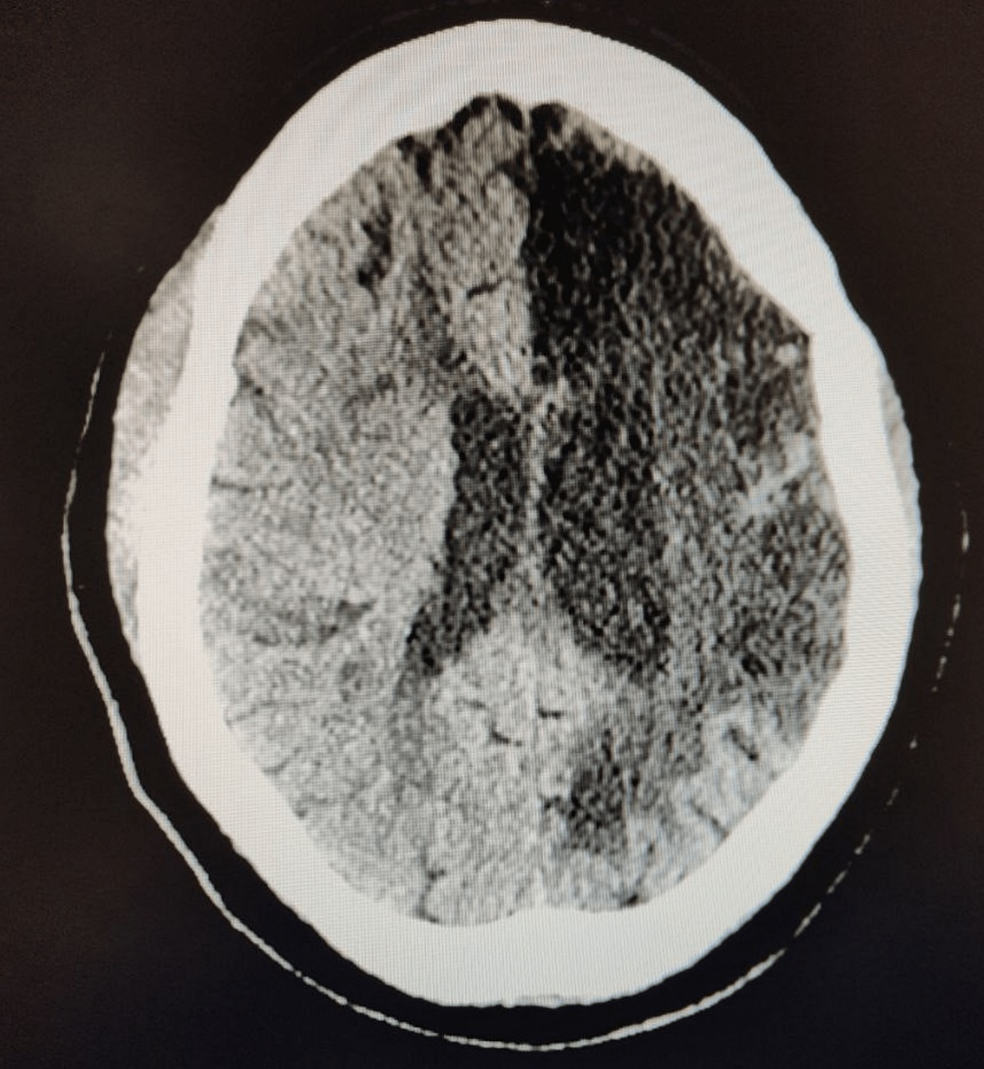
CT head without contrast showed no signs of acute hemorrhage. Diffuse hypodensity of the left hemisphere indicative of previous stroke.

Non-contrast CT of the head demonstrated no signs of acute hemorrhage and showed severe encephalomalacia of the left hemisphere, consistent with a previous L MCA stroke (Figure 1).

Laboratory investigations included a comprehensive metabolic panel and a complete blood count, which yielded normal results. Electrocardiography (EKG) showed normal sinus rhythm, and chest X-ray appeared normal. Two-dimensional echocardiography (2D echo) did not reveal any significant abnormalities.

**Figure 2:**
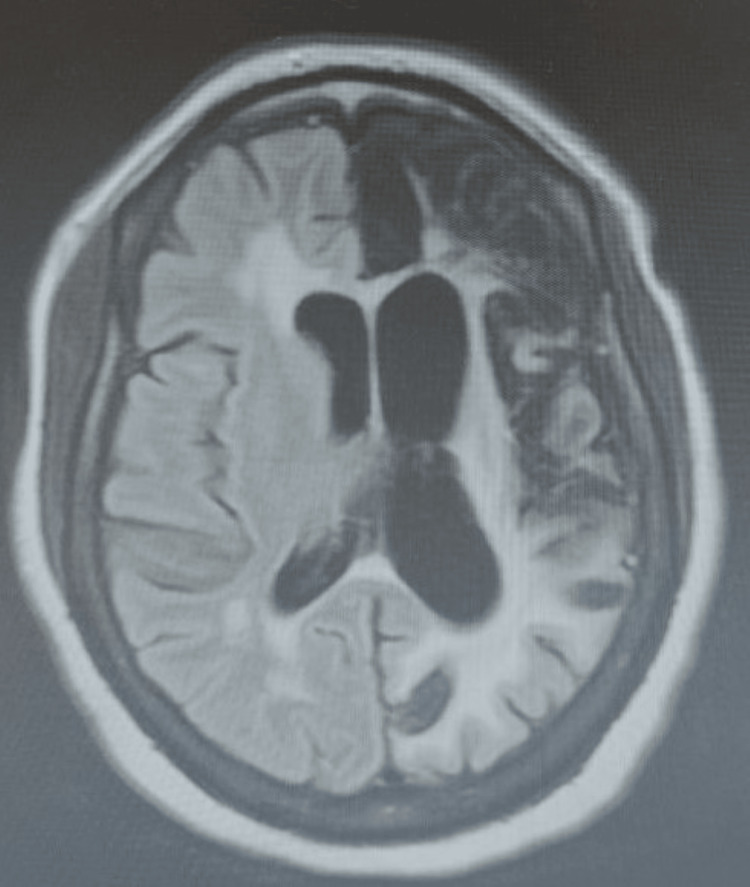
MRI FLAIR head demonstrates a large area of infarction in the left cerebral hemisphere with extensive white matter gliosis and associated ex vacuo dilatation of the lateral ventricle. MRI FLAIR: magnetic resonance imaging with fluid-attenuated inversion recovery

**Figure 3:**
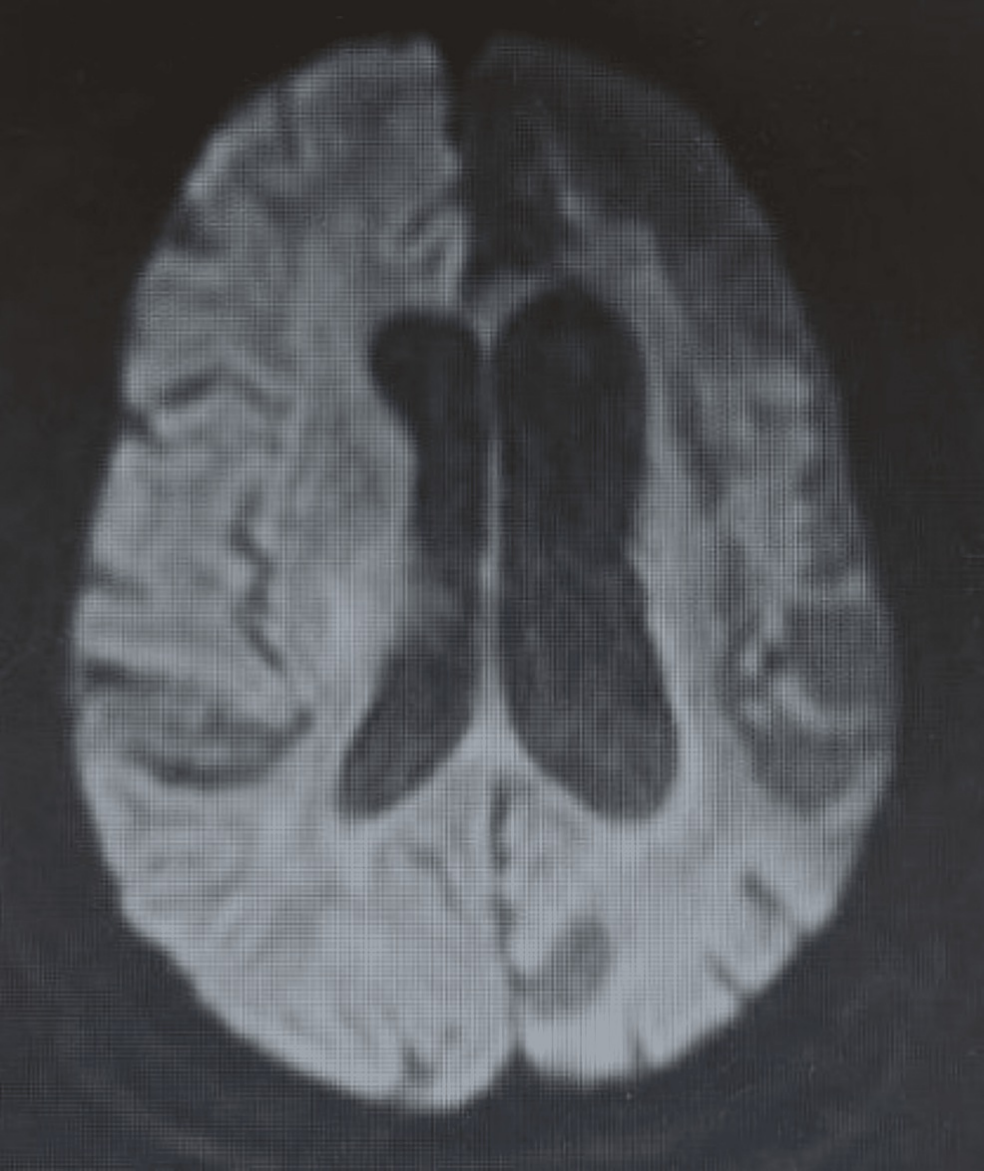
MRI DWI of the head shows no acute intracranial process. Large-volume encephalomalacia in the left cerebral hemisphere involving the ACA, MCA, and PCA territories. MRI DWI: magnetic resonance imaging with diffusion-weighted imaging; ACA: anterior cerebral artery; MCA: middle cerebral artery: PCA: posterior cerebral artery

Brain MRI showed no evidence of acute infarction but revealed signs of old stroke, that is, hypointensity on fluid-attenuated inversion recovery (FLAIR) and DWI (Figures 2, 3) with correlates on ADC in the left hemisphere. Electroencephalogram (EEG) demonstrated a normal study with no epileptic foci.

Upon admission, the patient was initiated on secondary stroke prevention measures and administered a combination of aspirin and dipyridamole. This caused the patient to complain of severe headache. With the patient’s history of migraine, the medication chart was cross-referenced, and the combination drug was stopped and replaced with plain aspirin. The patient reported relief from headaches after discontinuation of the combination drug. Overnight, she experienced complete resolution of neurological symptoms. The patient was subsequently discharged from the hospital.

## Discussion

Several mechanisms have been proposed for PSR. First, there may be altered drug uptake, distribution, and/or metabolism in diseased or abnormal areas of the brain, making the brain more susceptible to sedatives, especially gamma-amino butyric acid (GABA) receptor agonists [6]. Second, some redundancies in neuronal numbers or circuitry that characterizes the normal state are lost focally in a diseased brain. Even minimal impairment of the remaining functioning neurons may produce an exaggerated response [9]. Third, another theory based on an animal model states that after an episode of stroke, exposure to central nervous system (CNS) antigens due to compromise of the blood-brain barrier (BBB) or release of antigen into the bloodstream leads to CNS-specific lymphocytes. Later, when the body produces significant systemic inflammation in response to a systemic stressor, CNS-specific lymphocytes can be activated. This can lead to stroke-like symptoms localized to the area of the previous insult [5].

Certain demographic characteristics were associated with a higher risk of PSR, including being female, being African American, or identifying as belonging to "other" races. Patients with recrudescence also had higher frequencies of diabetes, dyslipidemia, smoking, and more severe neurologic deficits at the time of the initial stroke, indicating that these comorbidities and the severity of stroke may increase vulnerability to PSR [1].

PSR is characterized by the inability of the brain to adapt to systemic stressors and is always preceded by such stressors. By contrast, only 20% of TIA cases have an identified stressor [2]. Currently, stressors are categorized into five main groups: systemic inflammation (e.g., fever and infections), hypotension, metabolic factors (e.g., hyponatremia and hypokalemia), chemical insults (benzodiazepines, sedatives, and opioids), and physiological stress (insomnia, fatigue, and excessive sun exposure).

Notably, in the case of the patient discussed here, there appeared to be a correlation between elevated blood pressure and the occurrence of episodes of PSR. Further research is needed to better comprehend the specific stressors and their relationships with PSR, including investigating the potential associations between elevated blood pressure and the occurrence of PSR episodes.

Patients with PSR typically have a history of similar episodes. Thus, a comprehensive history that includes a detailed summary of the symptoms of the patient during the initial stroke is paramount for effectively managing such cases. This becomes especially relevant in cases like that presented here, where the patient's prior history of TIA further complicates the diagnostic process. This information can help determine the typical presentation of PSR symptoms in the patient, which can aid in the diagnosis. In addition, understanding which triggers are more likely to induce symptoms can guide recommendations for the patient to avoid such triggers in the future. Inaccurate identification of PSR as a TIA can lead to unwarranted modifications in patient management. It is imperative to prioritize preventive measures and prompt treatment of infections, hyponatremia, and other medical complications associated with PSR while also exercising caution in the use of benzodiazepines. It is imperative to prioritize preventive measures and prompt treatment of infections, hyponatremia, and other medical complications associated with PSR, while also exercising caution in the use of benzodiazepines.

In this particular patient, thrombolytic treatment was appropriately deferred because she presented outside the thrombolytic window. Fortunately, this decision was favorable. Within a comprehensive stroke center, it has been observed that 21% of patients who received intravenous tissue plasminogen activator (IV tPA) were subsequently determined not to have experienced a stroke [10]. Although these patients did not exhibit worsened outcomes, it is worth noting that individuals with PSR have a distinct vascular risk profile compared with other conditions that mimic stroke symptoms. Therefore, it is essential to conduct studies that investigate the potential adverse consequences of incorrect initial diagnosis and management of PSR. Given the urgent nature of stroke cases, which necessitates prompt judgment and decision-making, there is a demand for clinical decision-making tools that can assess the likelihood of PSR in patients.

Moreover, such misdiagnoses may lead to a cascade of costly procedures, such as vascular imaging, cardiac ultrasound, Holter monitoring, and blood tests, which not only increases expenses but also prolongs the patient's hospitalization.

In clinical practice, we differentiate TIA from PSR based on the presence of deficits, which are new compared to the old stroke. Data are required on the accuracy of this method as, theoretically, there can be overlap scenarios, where TIA can present with same deficits.

Enhanced awareness regarding the temporal resolution of symptoms and anticipated duration of hospitalization has the potential to optimize resource allocation. This can be facilitated by stressing the differentials to consider when a patient presents with stroke-like symptoms and not only on the recommended initial imaging and treatment of stroke, which will lead to improved outcomes in such patients. Future studies are needed to generate a validated predictor score for this unique phenomenon to identify who may or may not benefit from a recurrent stroke work-up.

## Conclusions

PSR is a frequently encountered but often overlooked clinical entity characterized by an acute recurrence of previously resolved stroke deficits. This case report highlights the challenges in diagnosing and managing PSR and the need for increased awareness among healthcare professionals. The patient presented with multiple episodes of PSR, which were initially misdiagnosed in the emergency department, leading not only to unnecessary testing and confusion regarding the appropriate management protocols but also to prescribing a medication that triggered her other coexisting condition, leading to increased discomfort for the patient. Differentiating PSR from other conditions that mimic stroke, such as TIAs, is crucial to avoid inappropriate interventions and provide targeted preventive measures. Understanding the mechanisms underlying PSR, including altered drug responses and immune activation, can aid in the development of effective treatment strategies.

Moreover, the accurate identification of PSR can have important implications for healthcare resource allocation. Misdiagnosis can lead to unnecessary procedures, prolonged hospitalization, and increased costs. By improving the awareness of PSR and its distinct vascular risk profile, healthcare providers can optimize patient care, prioritize preventive measures, and avoid unnecessary interventions. Future research efforts should focus on developing validated prediction scores to identify patients at risk of PSR and to guide appropriate diagnostic workup. Clinical decision-making tools can aid in assessing the likelihood of PSR and support prompt and accurate diagnoses. Ultimately, enhancing the understanding and recognition of PSR will contribute to improving patient outcomes, resource allocation, and cost-effective healthcare delivery.
